# Probing Nanorod Assembly and Dynamics in Polymer Nanocomposites in Equilibrium and Shear

**DOI:** 10.1021/acs.macromol.5c01005

**Published:** 2025-08-06

**Authors:** Phillip A. Taylor, Ting Ge, Thomas C. O’Connor, Gary S. Grest

**Affiliations:** † Department of Chemical Engineering, University of Virginia, Charlottesville, Virginia 22904, United States; ‡ Department of Chemistry and Biochemistry, 2629 University of South Carolina, Columbia, South Carolina 29208, United States; § Department of Materials Science and Engineering, 6612 Carnegie Mellon University, Pittsburgh, Pennsylvania 15213, United States; ∥ 1105 Sandia National Laboratories, Albuquerque, New Mexico 87123, United States

## Abstract

Coarse-grained molecular dynamics simulations are used to examine the structure and dynamics of nanorod assemblies in polymer melts under equilibrium and simple shear. We show that as the concentration of nanorods increases, there is a transition from an isotropic phase to a two-phase region in which the nanorods phase separate into a dilute phase and dense bundles of hexagonally packed nanorods. The onset of the two-phase region is below that predicted by Onsager theory, which we attribute to an effective increase in the diameter of nanorods due to a layer of polymer bound to the rod surfaces. Equilibrium simulations show that increasing polymer chain length *N* enhances nanorod bundling at fixed nanorod concentration. Simulations of systems undergoing simple shear show that flow enhances nanorod alignment and bundling relative to those of equilibrium systems with similar properties. Finally, simulations reveal that increasing nanorod length enhances nanorod alignment under shear at equivalent shear rates but not at equivalent Péclet numbers. Overall, our simulations highlight that polymer matrix-nanorod attraction (i.e., chemistry), shear rate, and matrix chain length are desirable design variables to control the structure of nanorod-containing soft materials under simple shear.

## Graphical abstract

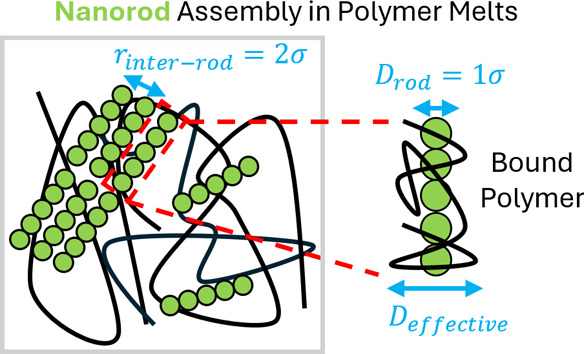

## Introduction

Nanorod fillers in polymer matrices have gained significant interest due to their enhanced optical,^1^ mechanical,^2^ and thermal properties^3^ compared to spherical particles. Despite recent advancements in understanding the equilibrium structure and properties of polymer nanocomposites,^4–10^ less is known about the structure, dynamics, and rheology of nanocomposites containing nanorods under simple shear. During processing of nanocomposites, polymers and nanoparticles are exposed to significant shear forces and mechanical deformation, which ultimately impact their structure and dynamics.^11^ Therefore, a fundamental understanding of the response of soft materials to nonequilibrium conditions is essential in designing soft materials with tailorable, viscoelastic properties.

Experimental studies of nanoparticles under shear flow include reports of both shear-induced particle dispersion and assembly.^12–16^ Therefore, it is unclear under which conditions dispersion or aggregation is favored. Experiments by Wong et al.^12^ showed insignificant effects of simple shear on the ordering of spherical assemblies of spherical, polymer-grafted nanoparticles, whereas shear flow induced the dispersion of anisotropic assemblies of spherical, polymer-grafted particles at low shear rates. At larger shear rates, smaller anisotropic particle assemblies coarsened into larger aggregates, reflecting the dominance of interparticle attractions over flow fields and thermal energy. Moreover, experiments by Gunes et al.^14^ and Scirocco et al.^15^ showed ordering of ellipsoidal and spherical colloidal particles, respectively, in shear-thinning polymer matrices under steady shear flow. In their work, Gunes et al.^14^ reported shear-induced clustering of ellipsoidal, hematite colloids in a matrix of high molecular weight, poly­(ethylene oxide) (PEO) for particle volume fractions of 0.1% < ϕ < 10.0%. As opposed to the necklace-like assembled structures seen for shear-induced clustering of spherical colloidal particles,^15^ Gunes et al.^14^ observed ellipsoidal particles forming sheet-like structures such that the assembled structures were elongated in the flow direction and parallel to the vorticity-gradient plane. Their microscopy images and corresponding fast Fourier transforms (FFTs) also revealed neighboring particles packed with an angle of approximately 30° between the lines joining their centers and the principal axis.

In addition to numerous experimental reports, recent molecular dynamics simulation studies have also examined the shear-induced assembly and disassembly of spherical nanoparticles^17^ and nanorods^18,19^ in polymer melts. Coarse-grained molecular dynamics simulation studies by Park et al.^19^ and Gao et al.^20^ of aggregating nanorods, such that nanoparticle–nanoparticle interactions mimic chemistries with attractive interactions between particles, have shown shear-induced dispersion with increasing shear rate. Conversely, simulation studies by Gao et al.^18^ of nonaggregating nanorods, such that nanoparticle–nanoparticle interactions mimic chemistries with repulsive interactions between particles, have reported enhanced nanorod alignment under flow, but insignificant effects of the shear rate on nanorod assembly and cluster sizes. Furthermore, molecular dynamics simulations have also been used to study nanorod assembly and orientation in melts containing diblock copolymers subject to simple shear flow.^21,22^ Dissipative particle dynamics (DPD) simulations by He et al.^21^ showed that the presence of selective nanorods, such that nanorods favored interactions with a single (A) block in the diblock copolymer, resulted in a shift in the critical shear rate to larger values, corresponding to the shear rate at which lamella structures underwent a transverse-to-perpendicular transition. Nonselective nanorods, corresponding to systems with equally repulsive interactions between nanorods and A and B polymer blocks, shifted the critical shear rate to lower values. Moreover, increasing the shear rate resulted in enhanced dispersion of nanorods in simulations of nonselective nanorods. A similar study by Pan et al.^22^ compared equilibrium and sheared morphologies of diblock copolymer-nanorod mixtures. Equilibrium systems formed cylindrical polymer assemblies, and shear simulations were performed using different shear axes relative to the cylinder, resulting in differences in packing between nanorods such that shear along the longitudinal axis of cylindrical polymer morphologies promoted the assembly of nanorods into a hexagonal lattice.

While the studies highlighted above have investigated the impact of simple shear flow on nanorod assembly and structure, an open area of research is the effect of shear flow on nanorod phase coexistence, specifically the effect of shear on the isotropic-to-nematic transition and two-phase regions under nonequilibrium conditions versus the corresponding transition for equilibrium systems predicted via Onsager theory.^23^ In this study, we use coarse-grained molecular dynamics simulations to examine the impact of polymer matrix chain length on the equilibrium and nonequilibrium phase behavior of polymer melts with nanorod fillers. First, we examine equilibrium systems and show that increasing polymer chain length promotes nanorod assembly and bundling due to nanorod-matrix chain bridging interactions for systems with attractive nanorod-matrix chain interactions. Next, we contrast the structures obtained from equilibrium simulations with those obtained from nonequilibrium simulations of simple shear. We observe that simple shear shifts the onset of nanorod bundling to lower nanorod volume fractions versus the corresponding equilibrium simulations. Increasing matrix chain length also shifts the two-phase region to lower nanorod volume fractions, and finally, we show that increasing nanorod length and nanorod volume fraction promote nanorod alignment with the flow direction for equivalent shear rates. At equivalent Péclet numbers, however, nanorod length and nanorod volume fraction show minimal effects on nanorod alignment.

## Models and Methods

### Simulation Models

In this work, we model nanorods and polymer matrix chains using the Kremer–Grest model.^24^ Coarse-grained beads have a mass of *m* and a size of σ. Nanorod bead–nanorod bead interactions are represented using a purely repulsive Lennard–Jones potential with a cutoff of *r*_c_ = 2^1/6^σ:

$$U_{\mathrm{LJ}}=\{\begin{array}{ll} 4\epsilon [(\sigma /r{)}^{12}-(\sigma /r{)}^{6}], & \mathrm{if}\;r\leq r_{c}\\ 0, & r>r_{c} \end{array}$$ 1

Matrix bead–matrix bead and nanorod bead–matrix bead interactions are modeled using attractive Lennard–Jones interactions with a cutoff of *r*_c_ = 2.5σ.

Monomers are connected via finitely extensible nonlinear elastic (FENE) bonds, with a spring constant of *k* = 30ϵ/σ^2^ and a maximum bond extension parameter of *R*_0_ = 1.5σ. Polymer matrix chains and nanorods have an average equilibrium bond length of 0.96σ. Chain stiffness is included via a 3-body bending potential *U*_θ_ = *k*_θ_(1 + cosθ). For the nanorods, we set *k*_θ_ = 1000ϵ to maintain rigid rod-like conformations, while the polymer matrix chains are modeled using *k*_θ_ = 1.5ϵ. With this very strong 3-body bending potential for the nanorods, our results agree with previous simulations of Wang et al.^25^ for the mobility of dilute nanorods in a polymer matrix. In this study, nanorods of lengths *l* = 8, 16, 32σ with diameter *d* = 1.0σ are simulated, and a schematic showing our nanorod model is shown in Figure 1a (right).

**Figure 1:**
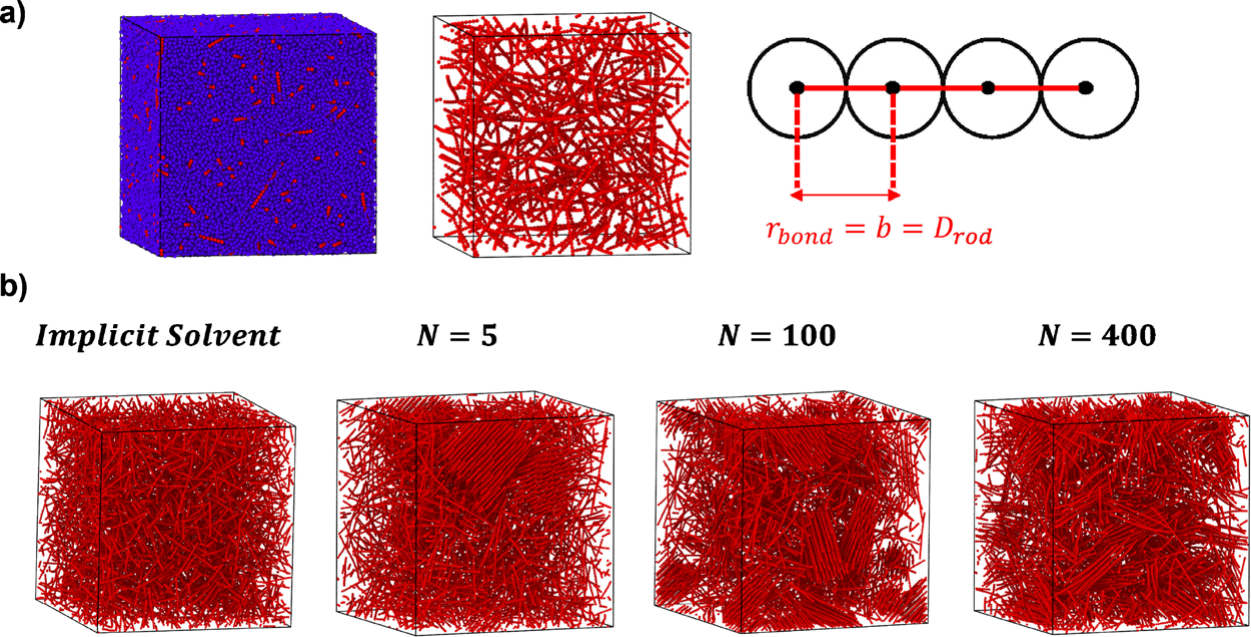
Schematic of our coarse-grained nanorod model and simulation snapshots from equilibrium simulations of nanorods (*l* = 32σ) at nanorod volume fractions of (a) ϕ_r_ = 3.6% and (b) ϕ_r_ = 7.0%. Snapshots shown in (a) left and middle show polymer matrix beads (blue) and nanorod beads (red). Matrix chains are omitted from (a) middle to show that nanorods are dispersed at ϕ_r_ = 3.6%. Snapshots of nanorods in part (b) are shown for an implicit good solvent and for varying polymer matrix chain length, *N*, (matrix chains not shown for clarity).

Systems containing nanorods in polymer melts are first equilibrated in the NPT ensemble with a Nosé–Hoover thermostat and barostat.^26^ Systems are maintained at a temperature of *T* = 1.0ϵ/*k*_B_ and a pressure of *P* = 0 in a cubic simulation box of length *L* with periodic boundary conditions in all three directions. Tables S1–S3 show system sizes (*L*) and nanorod volume fractions for all nanorod systems. The volume fraction of nanorods, ϕ_r_ = *N*_r_ *ld* ^2^/*L* ^3^, is varied between 0.02 and 24.1%, where *N*_*r*_ is the number of nanorods in a simulation box. The chosen temperature of *T* = 1.0ϵ/*k*_B_ is significantly greater than the glass transition temperature of the coarse-grained polymer matrix, *T*_g_ = 0.48ϵ/*k*_B_,^27,28^ such that we do not observe any glassy dynamics in this study. After equilibration, the simulations are then run at constant volume, and the thermostat is changed to a DPD thermostat,^29–31^ with a dissipative force constant ζ = 0.1*m*/τ and a cutoff *r*_c_ = 2^1/6^σ. Depending on *N* and *L*, simulations are run from 2 × 10^6^ to 9 × 10^6^τ. The time step for all simulations is 0.01τ, and all simulations are performed using LAMMPS.^32^

Shear is implemented by deforming the simulation cell using the *fix deform* command in LAMMPS.^32^ In this method, the angle between, say, the *x* and *y* direction is tilted at a constant rate. When the tilt reaches 45°, the cell is mapped to a tilt of – 45°, and the simulation continues. As the polymer beads are coupled to the pairwise DPD thermostat, which acts only between nearby beads, the velocity profile evolves without imposing a predetermined profile^33,34^ as with the standard SLLOD algorithm.^35^ This allows the system to form shear bands, which we observe for our longest chains (*N* = 400) for some shear rates, which the SLLOD algorithm does not, as it enforces a linear velocity profile in the shear plane. In this study, we perform simulations at shear rates of γ̇ from 10^–7^ to 10^–4^τ^–1^. As a reference time scale, the relaxation time of an entanglement strand in our coarse-grained model is τ_e_ = 1980τ.

Parallel and rotational diffusion coefficients are calculated using the methods of Wang et al.^25^ and Taylor et al.^36^ Briefly, the parallel component of the nanorod displacement is defined as the displacement of the center of mass of the nanorod along the rod axis of the body frame. Within a time interval Δ*t*, the unit vector along the nanorod changes from **u**(*t*) to **u**(*t* + Δ*t*). The center of mass displacement of the nanorod is defined as **s**(Δ*t*), and the parallel component as **s**_||_(Δ*t*) = [**s**(Δ*t*) · $\overset{-}{u(t)}$ ]**u**(*t*), where $\overset{-}{u(t)}$ is the mean of **u**(*t*) and **u**(*t* + Δ*t*). The displacement over time *t* along the rod axis, **s**_||_(*t*), is obtained by summing the parallel displacements in successive time intervals, and the parallel component of the mean-squared displacement (MSD) is determined as < Δ*r*_||_^2^(*t*) > = < *s*_||_^2^(*t*) >. The diffusion coefficient along the rod axis, *D*_||_, is determined as the long-time limit of < Δ*r*_||_^2^(*t*) > /2*t* such that the long-time value is calculated as the average value of < Δ*r*_||_^2^(*t*) > /2*t* for times greater than 10^5^τ.

To determine the rotational component of nanorod displacements, we use the methods of Kämmerer et al.^37,38^ The mean-squared angular displacements (MSAD) are unbounded, and the rotational diffusion coefficient is evaluated in the linear regime at large times. From *t* to *t* + Δ*t*, the angular displacement vector within a time interval Δ*t* is defined as $\phi \left(\Delta t\right)=\Delta \theta \frac{u\left(t\right)\times u\left(t+\Delta t\right)}{\left|u\left(t\right)\times u\left(t+\Delta t\right)\right|}$ , where Δθ = cos^–1^(**u**(*t*) ·**u**(*t* + Δ*t*)). The total angular displacement vector **ϕ**(*t*) is calculated by summing angular displacement vectors in successive time intervals, and the mean-squared angular displacement is therefore MSAD­(*t*) = <ϕ^2^(*t*)>. The rotational diffusion coefficient *D*_R_ is obtained as the long-time limit of MSAD/4*t* such that the long-time limit is calculated as the mean value of MSAD/4*t* for times greater than 10^5^τ.

To quantify nanorod alignment in equilibrium and under shear, we quantify the global and local nematic order parameters, *P*_2_ and *P*_2_^local^, respectively. For equilibrium simulations, the global nematic order parameter is defined as $P_{2}=\left\langle \frac{3}{2}\cos^{2}\theta -\frac{1}{2}\right\rangle$ , where θ is the angle between the end-to-end vectors of a pair of nanorods, and the ensemble average is performed over all pairs of nanorods and configurations. For the local nematic order parameter, $P_{2}^{\mathrm{local}}=\left\langle \frac{3}{2}\cos^{2}\theta -\frac{1}{2}\right\rangle$ , the ensemble average is performed between all pairs of nanorods that are separated by any inter-rod bead distance of 2.5σ or less. For nonequilibrium molecular dynamics simulations subject to simple shear flow, the angle θ in the definition of *P*_2_ is defined as the angle between the end-to-end vector of a nanorod and the shear axis such that the ensemble average is defined as the average value over all individual nanorods and configurations. Finally, we also compute nematic order parameters for polymer matrix chains *P*_2_^polymer^ subject to a simple shear flow. For polymer matrix chains, the angle θ is defined as the angle between a bond vector (i.e., the vector between two consecutive monomers on a matrix chain) and the shear axis, where the ensemble average is performed over all bond vectors across all matrix chains and configurations.

## Results and Discussion

### Equilibrium Structure and Assembly of Nanorods

Simulations of nanorods in polymer melts with nanorod volume fractions of ϕ_r_ ≤ 3.6% show dispersed systems with no nanorod bundling or alignment (Figure 1a) (left and middle). Nanorod systems with nanorod volume fractions of ϕ_r_ ≥ 7.0% (Figure 1b) exhibit nanorod bundling and local alignment of nanorods for matrix chain lengths of *N* = 5, 100, and 400. Increasing matrix chain length from unentangled (*N* = 5) to entangled (*N* = 100, 400) polymer matrix chains results in less dispersed systems and enhanced local alignment of nanorods. For nanorods of length *l* = 32σ and diameter *d* = 1σ, Onsager theory^23,39^ predicts a transition from an isotropic-to-nematic phase at a nanorod volume fraction of approximately ϕ_r,Onsager_ = 14.4%. In this study, we observe nanorod bundling and alignment at nanorod volume fractions significantly below the predicted Onsager transition.

We attribute the disagreement between the predictions of the Onsager theory for the isotropic-to-nematic transition and our simulation results for nanorods in a polymer matrix to an increase in the effective diameter of nanorods due to bound polymer chains resulting from attractive interactions between polymer matrix chains and nanorods in our coarse-grained model. In agreement with this hypothesis, simulation snapshots of nanorods in an implicit good solvent (Figure 1b left) show that the absence of attractive matrix chains eliminates nanorod bundling and alignment at equivalent nanorod volume fractions. Moreover, simulation snapshots confirm that nanorods in implicit solvent only form a nematic phase at nanorod volume fractions above the predicted Onsager transition, ϕ_r,Onsager_ (Figure S1). Upon nanorod bundling, implicit solvent systems show randomly packed structures with variable spacing between nanorods, in contrast to the well-defined hexagonal packing exhibited by nanorods in entangled melts with attractive interactions between rods and matrix chains (ϕ_r_ = 24.1%, Figure S1). Normalized probability distributions of inter-rod bead distances for systems with explicit matrix chains show the emergence of a primary, well-defined peak upon nanorod bundling (ϕ_r_ = 7.0%), corresponding to an inter-rod spacing of 2σ (Figure S2). The randomly packed structures for implicit solvent systems stem from a lack of trapped matrix chains within the nanorod bundles.

We note that the thickness of the bound polymer layer observed in the simulations is not an explicit parameter in our coarse-grained model. Rather, the layer thickness is set by the equilibrium melt density. After accounting for the rod radius, the inter-rod bead spacing of 2σ leaves a spacing of 1σ between rods, corresponding to a single bound polymer. Assuming a bound layer thickness of *r*_mat_ = 1σ, we use Onsager theory to calculate the isotropic-to-nematic transition that is consistent with the effective rod diameter (*d*_e_) such that the diameter includes contributions from both the rod diameter (*d*_rod_) and the bound polymer (*d*_e_ = *d*_rod_ + 2*r*_mat_ = 3σ). Therefore, the Onsager theory predicts an order–disorder transition at 4.3%, assuming a single bound polymer. This result is consistent with our simulations, which show that the isotropic-to-nematic transition occurs between nanorod volume fractions of 3.6 and 7.0%.

Next, to explore the impact of the presence of attractive polymer matrix chains on nanorod dynamics before the onset of nanorod bundling, we calculate parallel and rotational nanorod diffusion coefficients (Figure 2a,b). Increasing nanorod volume fraction shows a negligible effect on parallel and rotational nanorod diffusion for entangled polymer matrices (*N* = 100, 400) for dispersed nanorod systems (ϕ_r_ < 7.0%). In contrast, unentangled (*N* = 5) matrix chains show a decrease in parallel and rotational nanorod diffusion coefficients with increasing nanorod volume fraction for dispersed nanorod systems. Our simulation results are in qualitative agreement with coarse-grained molecular dynamics simulations by Gao et al.^40^ of nanorods in melts of short (*N* = 30) polymer matrix chains. Their simulations showed a decrease in nanorod (bead) diffusion coefficients with increasing nanorod volume fraction between nanorod volume fractions of ϕ_r_ = 5.0–15.0%, followed by an increase in diffusion coefficients with increasing nanorod volume fractions of ϕ_r_ = 15.0–25.0%. We observe a similar decrease in the nanorod diffusion coefficients for short, unentangled matrix chains (*N* = 5); however, we are unable to report nanorod diffusion coefficients for volume fractions ϕ_r_ ≥ 7.0*%*. The mean-squared displacements (MSDs) and mean-squared angular displacements (MSADs) for all nanorod systems as a function of matrix chain length (*N*) and nanorod volume fraction (ϕ_r_) are shown in Figures S3 and S4. Furthermore, in agreement with previous reports,^25,36^ we observe a decrease in parallel and rotational nanorod diffusion coefficients with increasing nanorod length for dispersed systems for nanorod lengths of *l* = 8, 16, 32σ (Figure S5).

**Figure 2:**
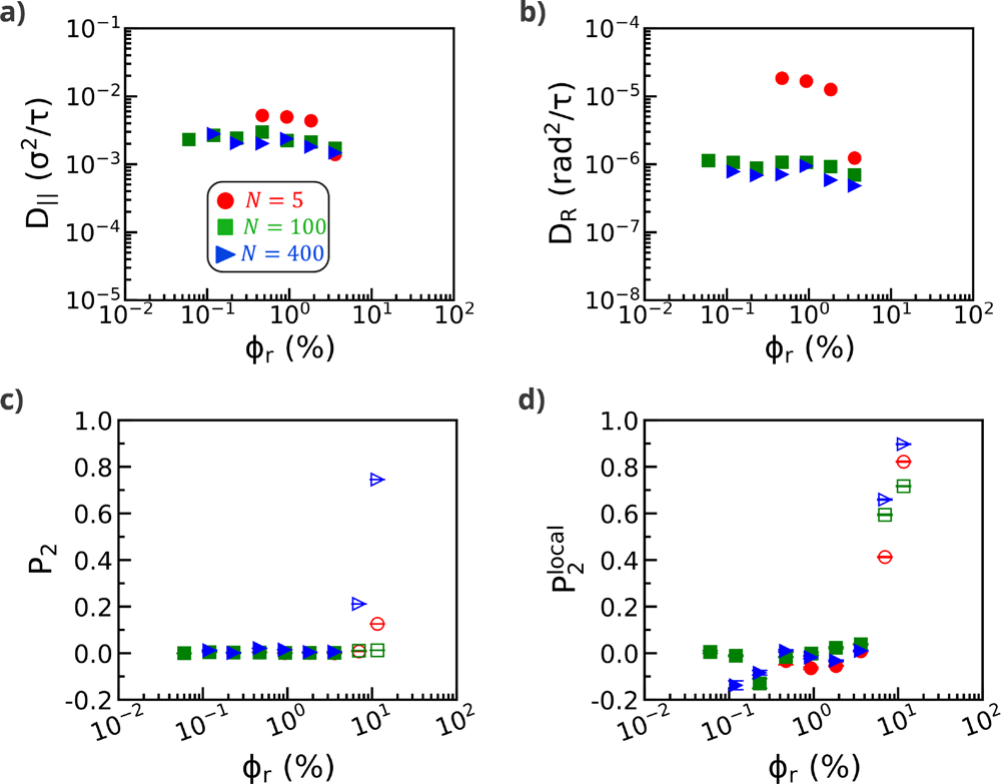
Nanorod diffusion coefficients and global *P*_2_ and local *P*_2_^local^ values versus nanorod volume fraction ϕ_r_ for a fixed nanorod length of *l* = 32σ. Parallel (a) and rotational (b) diffusion coefficients are shown for polymer matrix chain lengths of *N* = 5, 100, and 400. Global (c) and local (d) *P*_2_ values are shown for dispersed (closed symbols) and bundled (open symbols) nanorod systems.

In addition to the structural and diffusion analyses discussed above, simulations also reveal differences in nanorod alignment with varying nanorod volume fractions for dispersed and bundled nanorod systems. Specifically, dispersed nanorod systems show negligible nanorod alignment as measured via global and local nematic order parameters (*P*_2_ and *P*_2_^local^, respectively) of approximately 0 for nanorod volume fractions of ϕ_r_ < 7.0% (Figure 2c,d). Nanorod bundling and alignment occur for nanorod volume fractions of ϕ_r_ ≥ 7.0%, corresponding to an increase in global and local nematic order parameters. Upon nanorod bundling, simulations show that nanorod systems with the longest matrix chains (*N* = 400) promote greater global nanorod alignment (high *P*_2_) versus shorter matrix chains (*N* = 5, 100) (Figure 2c). We attribute this result to attractive bridging interactions between nanorods and polymer matrix chains, which facilitate nanorod bundling and global alignment for longer matrix chains. On smaller length scales (*r* < 2.5σ), however, matrix chain length *N* has a lesser effect on the local alignment of nanorods, as shown by similar local nematic order parameters for *N* = 5, 100, 400 (Figure 2d).

### Structure and Assembly of Nanorods under Simple Shear Flow

To elucidate the impact of simple shear flow fields on nanorod assembly and dynamics in contrast to the equilibrium results presented above, we perform nonequilibrium molecular dynamics simulations of nanorods in polymer melts. Subject to simple shear, dilute systems of nanorods in polymer melts (ϕ_r_ ≤ 0.9%) show alignment of nanorods with the flow direction but show no signs of nanorod bundling (Figure 3a right). Corresponding equilibrium simulations of nanorods for dilute systems are significantly below the predicted Onsager transition and do not show signatures of nanorod bundling or assembly (Figure 3a left). While equilibrium simulations show nanorod bundling and alignment at ϕ_r_ ≥ 7.0%, shear shifts the onset of nanorod bundling to lower nanorod volume fractions (ϕ_r_ ≥ 1.8%) (Figure 3b right). Nanorods assemble to form bundles in a hexagonal lattice with a well-defined spacing between rods. Due to the attractive Lennard–Jones interactions between nanorods and matrix chains, upon nanorod bundling, there are matrix chains located in the spacing between nanorods within bundles. Therefore, the underlying driving force for nanorod bundling in such systems stems from attractive bridging interactions between nanorods and matrix chains, resulting in an increase in the effective diameter of the rods due to the bound polymer matrix chains.

**Figure 3:**
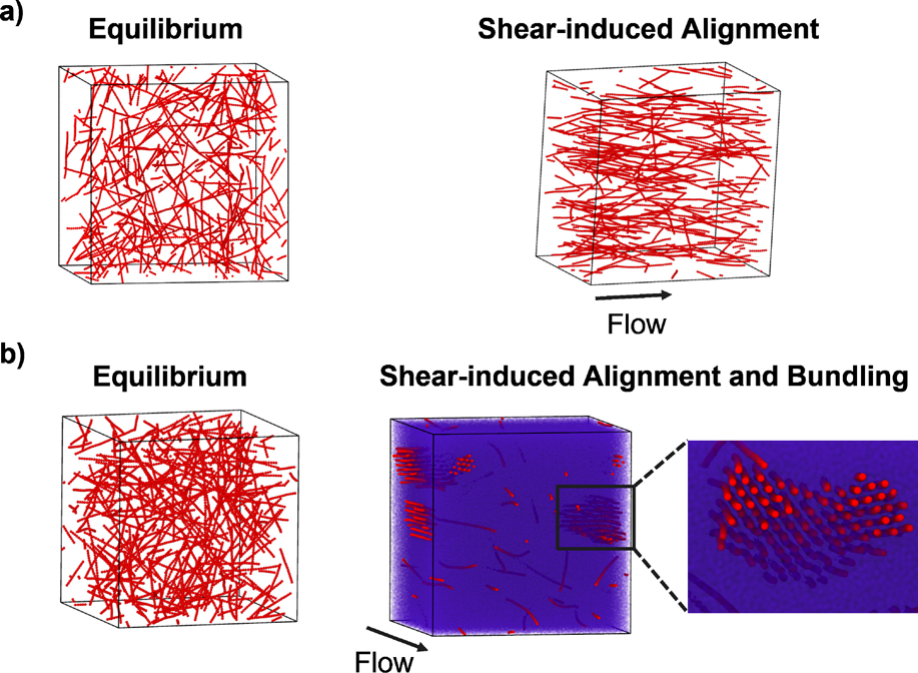
Visualizations of nanorods in polymer melts at equilibrium and subjected to simple shear. Snapshots are shown at nanorod volume fractions of (a) ϕ_r_ = 0.9% and (b) ϕ_r_ = 1.8%. Nanorod beads are shown in red, and polymer matrix chain beads are in purple. For clarity, polymer matrix chains are omitted for all snapshots except part (b) right. Data are shown for nanorods of length *l* = 32σ and matrix chain length of *N* = 100. Simulation snapshots showing simple shear correspond to a shear rate of γ̇ = 10^–4^τ^–1^.

Previous simulation studies have reported insignificant effects of the shear rate on the maximum cluster size and number of clusters of short nanorods (*l* = 5σ) in polymer melts with attractive nanorod-matrix chain interactions.^18^ Similarly, we do not observe nanorod bundling under shear for systems containing shorter nanorods (*l* ≤ 16σ) for the shear rates and nanorod volume fractions explored in this study. Therefore, the data suggest that there exists a critical nanorod volume fraction for nanorod bundling under shear, which is dependent on nanorod length. The relationship among the bundling transition, nanorod length and diameter, and polymer melt environment (nanorod-matrix attraction strength and matrix chain length) is beyond the scope of this study. Moreover, other simulation studies report shear-induced dispersion instead of shear-induced assembly for nanorods^19^ and spherical nanoparticles^17^ in polymer melts; however, such studies examined self-attracting nanoparticles and nanorods (systems with attractive Lennard–Jones interactions between nanorods), whereas our study examines nanorods with repulsive nanorod–nanorod interactions.

Next, we quantify the spacing between nanorods within bundles that are assembled under shear flow (Figure 4a,b) via probability distributions of perpendicular (gradient-vorticity plane) inter-rod bead distances (*P*(*r*_⊥_)). Dispersed systems of nanorods under shear flow (ϕ_r_ ≤ 0.9%) exhibit monotonically increasing distributions of distances with increasing *r*_⊥_, ranging from a monomer size (σ) to large distances (*r*_⊥_ ≥ 20σ), with no preference for close inter-rod bead contacts (Figure 4a). Upon nanorod bundling (ϕ_r_ ≥ 1.8%) (Figure 4b), we observe probability distributions with a first peak located at approximately 2σ, indicating a preference for close inter-rod bead contacts with well-defined spacing between rods, similar to the inter-rod spacing observed for equilibrium systems (Figure S2). An inter-rod spacing of 2σ allows for interpenetration of matrix chains within nanorods bundles, in agreement with simulation snapshots of sheared nanorod systems (Figure 3b right). Polymer matrix chains that are wedged between nanorods are also aligned with the flow direction (Figure 3b). Nematic order parameters (*P*_2_^polymer^) show an increase in polymer matrix chain alignment upon nanorod bundling (ϕ_r_ ≥ 1.8%) (Figure 4c), mirroring results from simulation snapshots. The magnitudes of polymer nematic order parameters (0.00 < *P*_2_^polymer^ < 0.16), however, are significantly smaller than those seen for nanorods (Figure 2c,d) due to (a) our definition of *P*_2_^polymer^, which uses bond vectors to define orientation vectors along the polymer backbone, and (b) the small fraction of polymer chain segments that are trapped within nanorod bundles as compared to the bulk (free) chain segments for long, entangled polymers, which have contour lengths much greater than the average nanorod bundle size.

**Figure 4:**
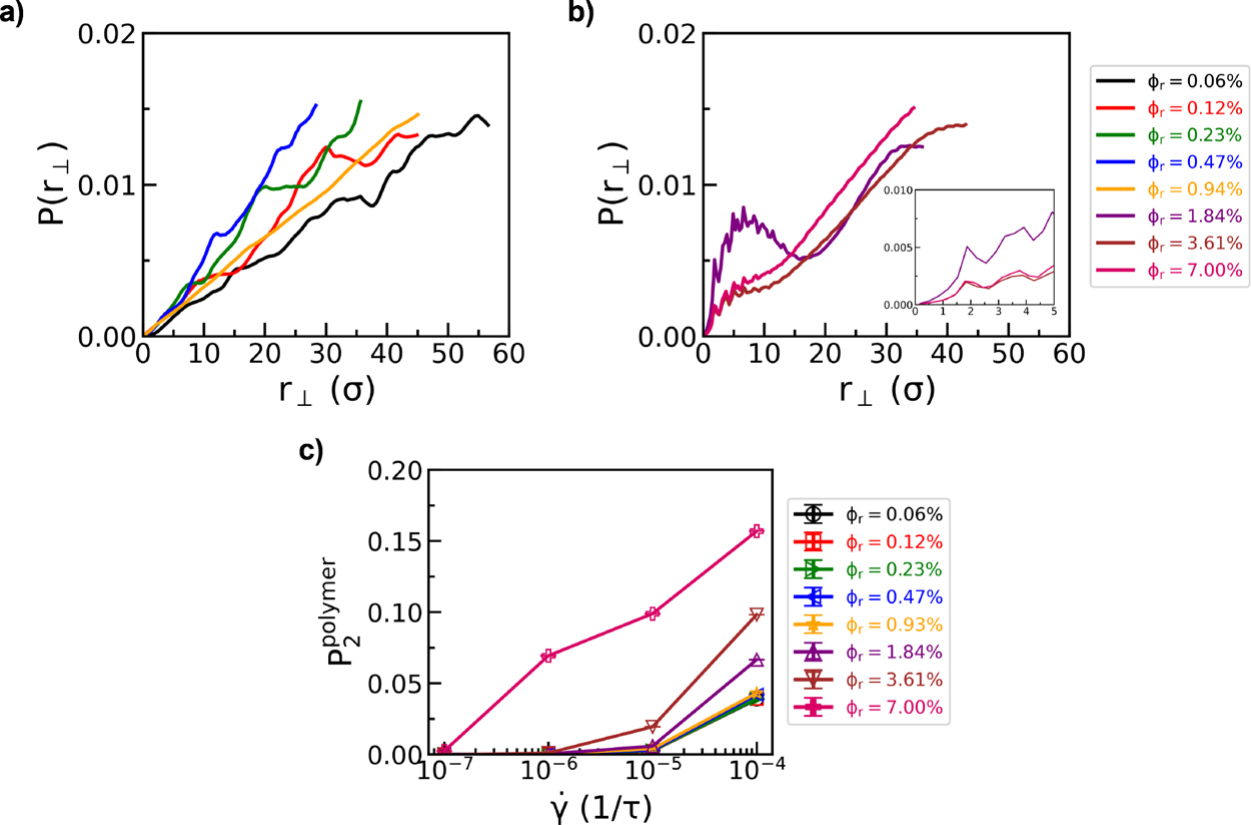
Probability distributions of perpendicular (gradient-vorticity plane) inter-rod bead distances, *P*(*r*_⊥_), for (a) dispersed and (b) bundled nanorod systems as a function of nanorod volume fraction, ϕ_r_, for a shear rate of γ̇ = 10^–4^τ^–1^. Inset: zoomed-in plot of *P*(*r*_⊥_) for 0 ≤ *r*_⊥_ ≤ 5σ. (c) Nematic order parameters for polymer matrix chains, *P*_2_^polymer^ as a function of γ̇ and ϕ_r_. All probability distributions in (a,b) are probability mass functions. Data are shown for a polymer matrix with a chain length of *N* = 100.

The results shown above highlight similar mechanisms of nanorod bundling via matrix chain bridging for both equilibrium and nonequilibrium nanorod systems. Given that equilibrium systems exhibit nanorod assembly that depends sensitively on matrix chain length, next we explore the impact of increasing matrix chain length on nanorod bundling in entangled polymer matrix chains under shear flow. Simulation snapshots of nanorods in polymer melts under shear (Figure 5) for matrix chain lengths of *N* = 100 and 400 are shown for similar Weissenberg numbers, *Wi* = τ_e_ *Z* ^2^γ̇, where τ_e_ = 1980τ is the relaxation time of an entanglement strand, *Z* = *N*/*N*_e_ is the number of entanglements per matrix chain, and *N*_e_ = 28 for our coarse-grained model. Snapshots are shown at *Wi* = 2.5 for *N* = 100 (γ̇ = 10^–4^τ^–1^) and *Wi* = 4.0 for *N* = 400 (γ̇ = 10^–5^τ^–1^). Similar to equilibrium systems, increasing matrix chain length promotes nanorod bundling and alignment across multiple nanorod volume fractions ϕ_r_ = 0.9–7.0%. Snapshots also show that increasing the matrix chain length from *N* = 100 to *N* = 400 shifts the onset of nanorod bundling to lower nanorod volume fractions (ϕ_r_ = 0.9%). Furthermore, simulations of nanocomposites with matrix chains of length *N* = 400 reveal the existence of shear banding at shear rates of γ̇ = 10^–4^ and 10^–5^τ^–1^, as shown in Figure 6, while the homopolymer melt with no nanorods does not show a shear band for γ̇ = 10^–4^. Nanorod density profiles (γ̇ = 10^–4^) also show that nanorods partition into the regions where the velocity is constant.

**Figure 5:**
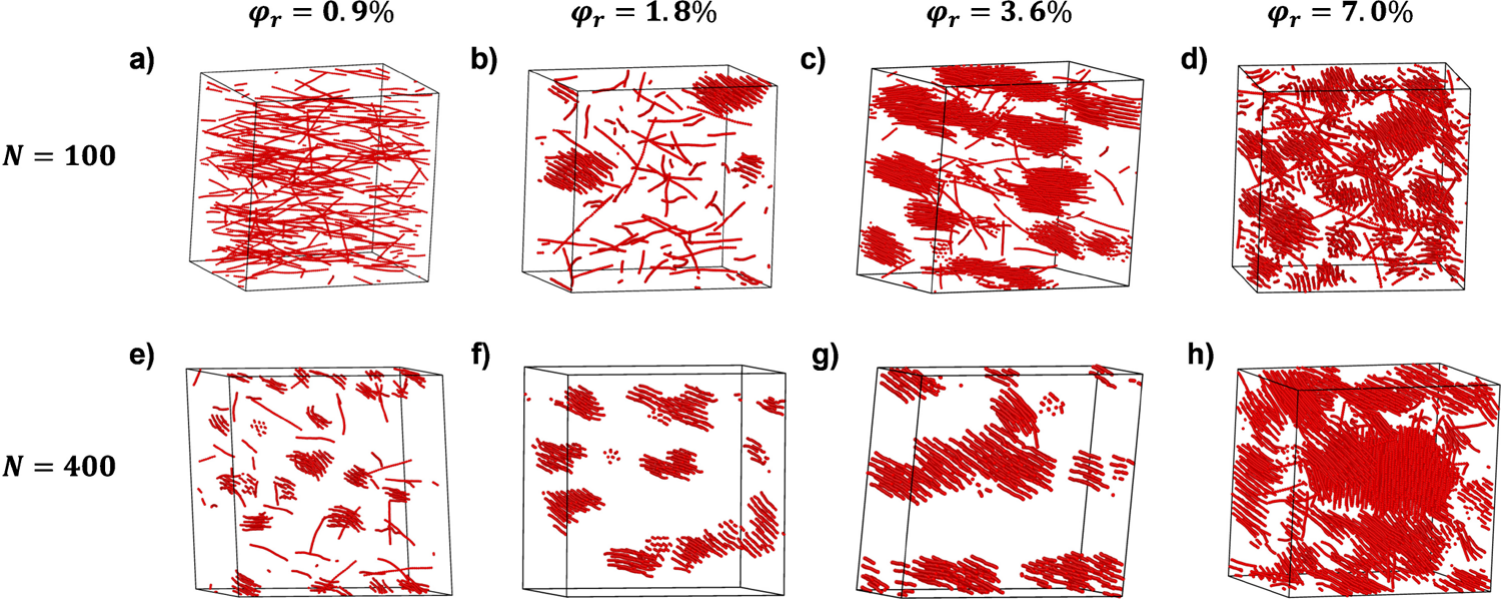
Simulation snapshots of nanorods (*l* = 32σ) under shear at nanorod volume fractions of (a,e) ϕ_r_ = 0.9%, (b,f) ϕ_r_ = 1.8%, (c,g) ϕ_r_ = 3.6%, and (d,h) ϕ_r_ = 7.0%. Snapshots are shown for matrix chain lengths of (a–d) *N* = 100 at a Weissenberg number *Wi* = 2.5 (γ̇ = 10^–4^τ^–1^) and (e–h) *N* = 400 at *Wi* = 4.0 (γ̇ = 10^–5^τ^–1^). Polymer matrix chains are omitted for clarity, and only nanorods are shown.

**Figure 6:**
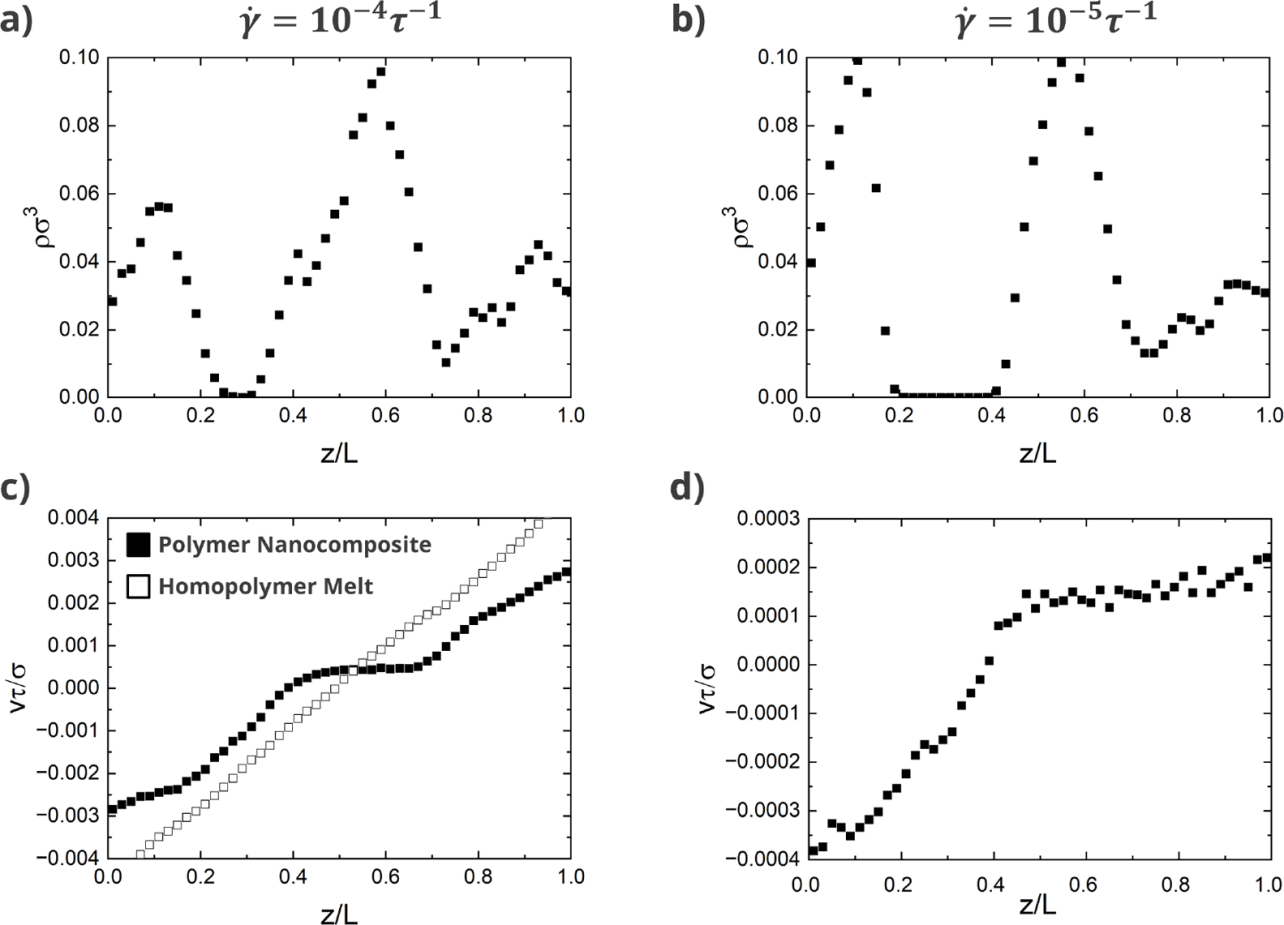
Nanorod density and velocity profiles for polymer melts with matrix chains of length *N* = 400, nanorod volume fraction of ϕ_r_ = 3.6%, and shear rates of (a,c) γ̇ = 10^–4^τ^–1^ and (b,d) γ̇ = 10^–5^τ^–1^ showing the existence of shear bands. Solid symbols on all subfigures correspond to systems with nanorods of length *l* = 32σ in polymer melts. Open symbols correspond to pure homopolymer melt with no nanorods.

In this work, we note that the ordered domains incorporate matrix chains such that they behave like grafted particles. It is unclear whether this eliminates or creates new effective entanglements. Coarse-grained simulations of spherical nanoparticles in polymer melts by Li et al. showed that upon increasing nanoparticle volume fractions from 0 to 42%, the matrix chains gradually disentangled as measured by a decrease in the number of kinks (entanglements) per chain.^41^ Nonequilibrium molecular dynamics simulations of homopolymer melts also showed an increase in entanglement length under shear.^42–44^ Therefore, future work will focus on the effects of nanorod volume fraction and shear on polymer entanglements.

Finally, we examine the impact of the nanorod volume fraction and nanorod length on the alignment of nanorods in entangled polymer matrix chains under shear flow. As expected, increasing nanorod volume fraction results in enhanced nanorod alignment (high *P*_2_) under simple shear flow (Figure 7a) for nanorods of length, *l* = 32σ. Decreasing nanorod length to *l* = 16σ (Figure 7b) and *l* = 8σ (Figure 7c) leads to a reduction in nanorod alignment and weaker dependence of nanorod alignment on nanorod volume fraction. While longer nanorods show a preference for alignment during shear flow, longer rods are also biased to remain aligned along the flow direction due to a reduction in nanorod rotational diffusion coefficients (*D*_R_) with increasing nanorod length (Figure S5). To account for differences in nanorod rotational diffusion coefficients, we investigate the dependence of nanorod alignment on the Péclet number. The Péclet number is defined as *Pe* = γ̇/*D*_R_ such that the advective transport (γ̇) of the nanorods is appropriately normalized by their diffusive transport (*D*_R_), therefore accounting for differences in diffusivities as a function of nanorod length and nanorod volume fraction. Simulations show that nanorod alignment as measured via *P*_2_ shows no dependence on the nanorod length as a function of the Péclet number (Figure 7d). Moreover, *P*_2_ values show minimal dependence on nanorod volume fraction when normalized via the Péclet number, in qualitative agreement with experiments by Calabrese et al.^45^ on colloidal rods in crowded polymer solutions.

**Figure 7:**
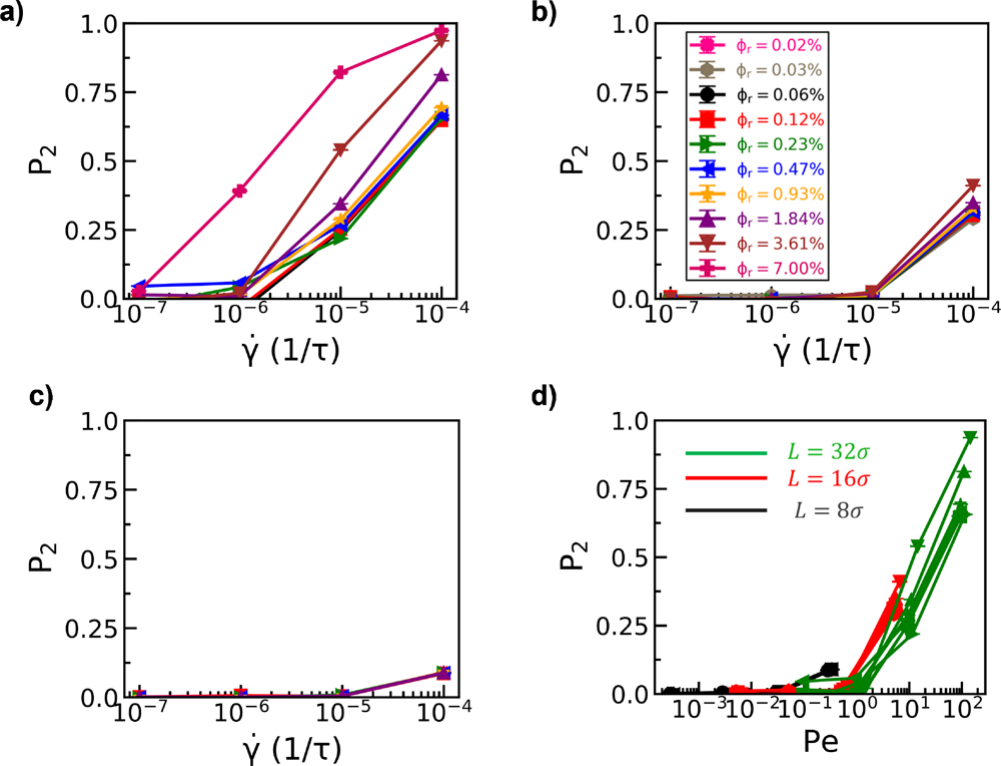
Nematic order parameters *P*_2_ of nanorods under shear flow in entangled polymer matrix chains of chain length *N* = 100 as a function of nanorod length, shear rate γ̇, and Péclet number. The legend shown in part (b) is applicable to subfigures (a), (b), and (c). Data are shown for nanorod lengths of (a) *l* = 32σ, (b) *l* = 16σ, and (c) *l* = 8σ. (d) *P*_2_ values are shown as a function of the Péclet number, where the Péclet number is defined as *Pe* = γ̇/*D*_R_, where *D*_R_ is the rotational diffusivity for a given nanorod length and nanorod volume fraction.

## Conclusions

Coarse-grained molecular dynamics simulations were used to investigate the effect of polymer matrix chain length and nanorod length on the assembly of nanorods in polymer melts in equilibrium and simple shear. Equilibrium simulations showed local nanorod alignment and bundling at nanorod volume fractions below the predicted Onsager transition. The shift in the bundling transition was attributed to an increase in the effective diameter of nanorods due to attractive bridging interactions between nanorods and polymer matrix chains. Moreover, at equilibrium, nanorod volume fraction had an insignificant effect on nanorod parallel and rotational diffusion coefficients in entangled melts for dispersed nanorod systems below the isotropic-to-nematic transition. Increasing the matrix chain length promoted global nanorod alignment but had an insignificant effect on local nanorod alignment.

Under simple shear, nanorods assembled to form bundles at nanorod volume fractions lower than those of the corresponding equilibrium simulations. Upon nanorod bundling, nanorods assembled in a hexagonal lattice, with a lattice spacing of approximately two monomer (σ) sizes, thus allowing for interpenetration of polymer matrix chains within nanorod bundles. Moreover, interpenetrating polymer matrix chains became more aligned along the shear direction with an increasing nanorod volume fraction. Interestingly, increasing matrix chain length promoted nanorod bundling both at equilibrium and under simple shear at equivalent Weissenberg numbers. Increasing the nanorod volume fraction also resulted in enhanced nanorod alignment along the shear direction, and longer nanorods showed a higher propensity for alignment along the shear direction versus shorter rods as a function of shear rate. At a constant Péclet number, however, nanorod length and nanorod volume fraction had an insignificant effect on nanorod alignment, in agreement with previously published experimental studies. Overall, this work highlights the impact of matrix chain molecular weight and nanorod size on the phase behavior of polymer nanocomposites both at equilibrium and nonequilibrium conditions. Therefore, the results of this study will guide the design of novel nanocomposite materials that can be utilized under a broad range of processing conditions and varying polymer matrix molecular weights and chemistries.

## Supplementary Material



## Notes

Simulation parameters and system sizes, simulation snapshots of nanorod systems, mean-squared displacements, mean-squared angular displacements, and parallel nanorod diffusion coefficients (PDF)
