# Supplementary material for: Probing Nanorod Assembly and Dynamics in Polymer Nanocomposites in Equilibrium and Shear
Source: Macromolecules. 2025 Aug 6;58(16):8698–706. doi: 10.1021/acs.macromol.5c01005 (PMC12392741; doi:10.1021/acs.macromol.5c01005)
Supplement: Supplementary file 1 [file ma5c01005_si_001.pdf]

Electronic Supporting Information: Probing Nanorod Assembly and Dynamics in Polymer Nanocomposites in Equilibrium and Shear

Phillip A. Taylor,*,† Ting Ge,‡ Thomas C. O'Connor,¶ and Gary S. Grest§

†*Department of Chemical Engineering, University of Virginia, Charlottesville, Virginia 22904, USA*

‡*Department of Chemistry and Biochemistry, University of South Carolina, Columbia, South Carolina 29208, USA*

¶*Department of Materials Science and Engineering, Carnegie Mellon University, Pittsburgh, Pennsylvania 15213, USA*

§*Sandia National Laboratories,Albuquerque, New Mexico 87123 USA*

E-mail: rbh2vu@virginia.edu

Table S1: Parameters for simulations of nanorods in polymer melts. Data is shown for matrix chain length N, number of matrix chains N_c, number of nanorods N_r, simulation box size L, and nanorod volume fraction ϕ_r. Data is shown for a nanorod length of $l = 32\sigma$.

N	N_c	N_r	L (σ)	ϕ_r (%)
100	13000	27	113.7	0.06
100	6500	27	90.3	0.12
100	3250	27	71.7	0.23
100	1625	27	57.0	0.47
100	6500	216	90.6	0.94
100	3250	216	72.2	1.84
100	12992	1728	115.3	3.60
100	6464	1728	92.5	7.00
100	3200	1728	75.4	11.60
100	1600	1728	61.2	24.10
400	1600	27	89.7	0.12
400	800	27	71.2	0.23
400	400	27	56.5	0.47
400	1600	216	89.9	0.94
400	800	216	71.6	1.84
400	400	216	57.3	3.60
400	1600	1728	226.2	7.00
400	405	729	58.9	11.60

Table S2: Parameters for simulations of nanorods in polymer melts. Data is shown for matrix chain length N, number of matrix chains N_c, number of nanorods N_r, simulation box size L, and nanorod volume fraction ϕ_r. Data is shown for a nanorod length of $l = 16\sigma$.

N	N_c	N_r	L (σ)	ϕ_r (%)
100	13000	27	113.7	0.03
100	6500	27	90.3	0.06
100	3250	27	71.7	0.12
100	1625	27	57.0	0.23
100	6500	216	90.6	0.47
100	3250	216	72.2	0.94
100	12992	1728	115.3	1.84
100	6464	1728	92.5	3.60

Table S3: Parameters for simulations of nanorods in polymer melts. Data is shown for matrix chain length N, number of matrix chains N_c, number of nanorods N_r, simulation box size L, and nanorod volume fraction ϕ_r. Data is shown for a nanorod length of $l = 8\sigma$.

N	N_c	N_r	L (σ)	ϕ_r (%)
100	13000	27	113.7	0.015
100	6500	27	90.3	0.030
100	3250	27	71.7	0.060
100	1625	27	57.0	0.120
100	6500	216	90.6	0.230
100	3250	216	72.2	0.470
100	12992	1728	115.3	0.940
100	6464	1728	92.5	1.840

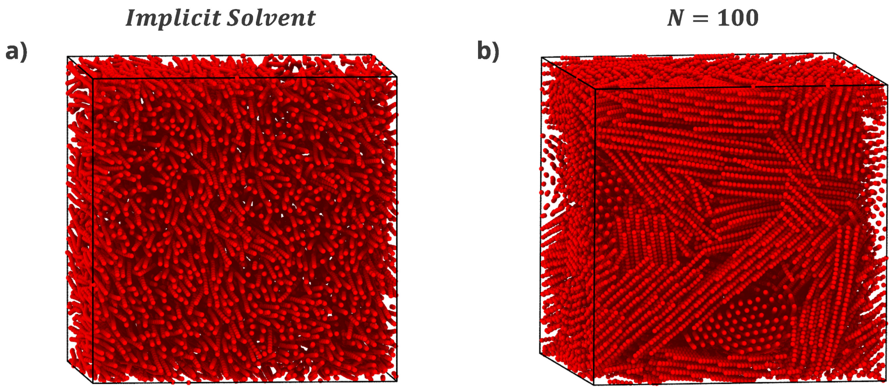

Figure S1: Simulation snapshots obtained from coarse-grained molecular dynamics simulations of nanorods of length $l = 32\sigma$ in (a) implicit solvent and (b) polymer melts with a matrix chain length, $N = 100$. Snapshots are shown at a nanorod volume fraction of $\phi_r = 24.1\%$

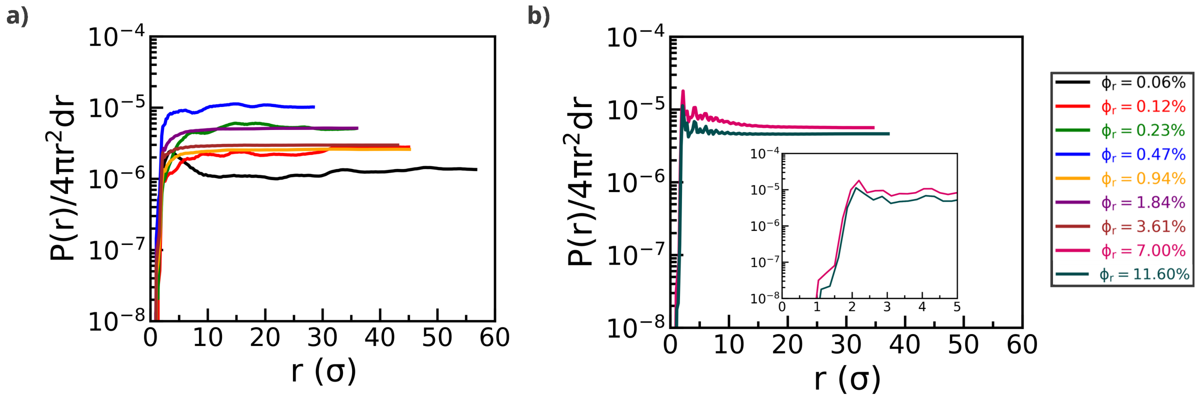

Figure S2: Normalized probability distributions of inter-rod bead distances, $P(r)/4\pi r^2 dr$, of nanorods of length $l = 32\sigma$ in a polymer matrix of matrix chain length $N = 100$ for (a) dispersed and (b) bundled nanorod systems as a function of nanorod volume fraction, ϕ_r, obtained from equilibrium molecular dynamics simulations. Inset: Zoomed-in plot of $P(r)/4\pi r^2 dr$ for $0 \leq r \leq 5\sigma$.

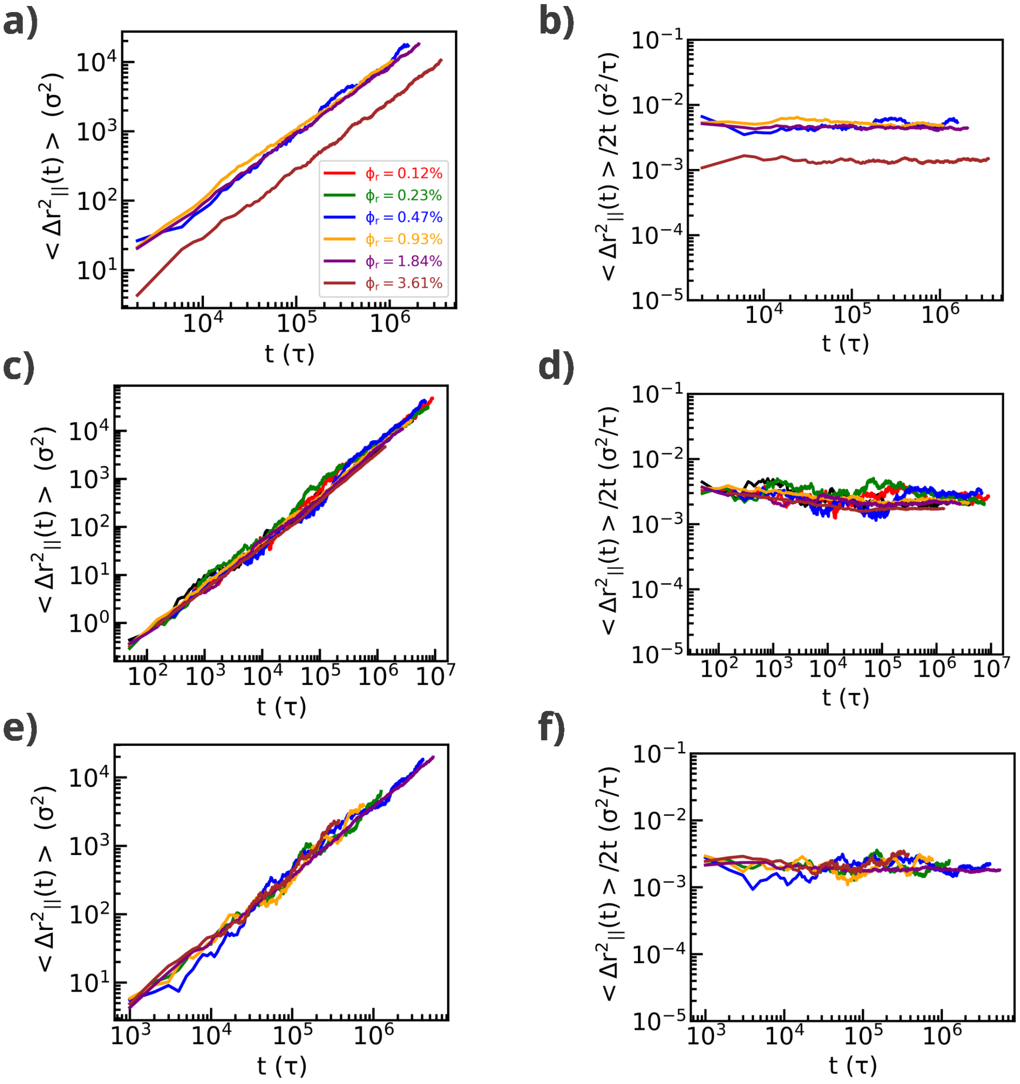

Figure S3: Parallel components of mean squared displacements (MSDs) $< \Delta r_{||}^2(t) >$ and $< \Delta r_{||}^2(t) > /2t$ as a function of nanorod volume fraction ϕ_r for nanorods of length $l = 32\sigma$ in polymer matrices of matrix chain length (a,b) $N = 5$, (c,d) $N = 100$, and (e,f) $N = 400$.

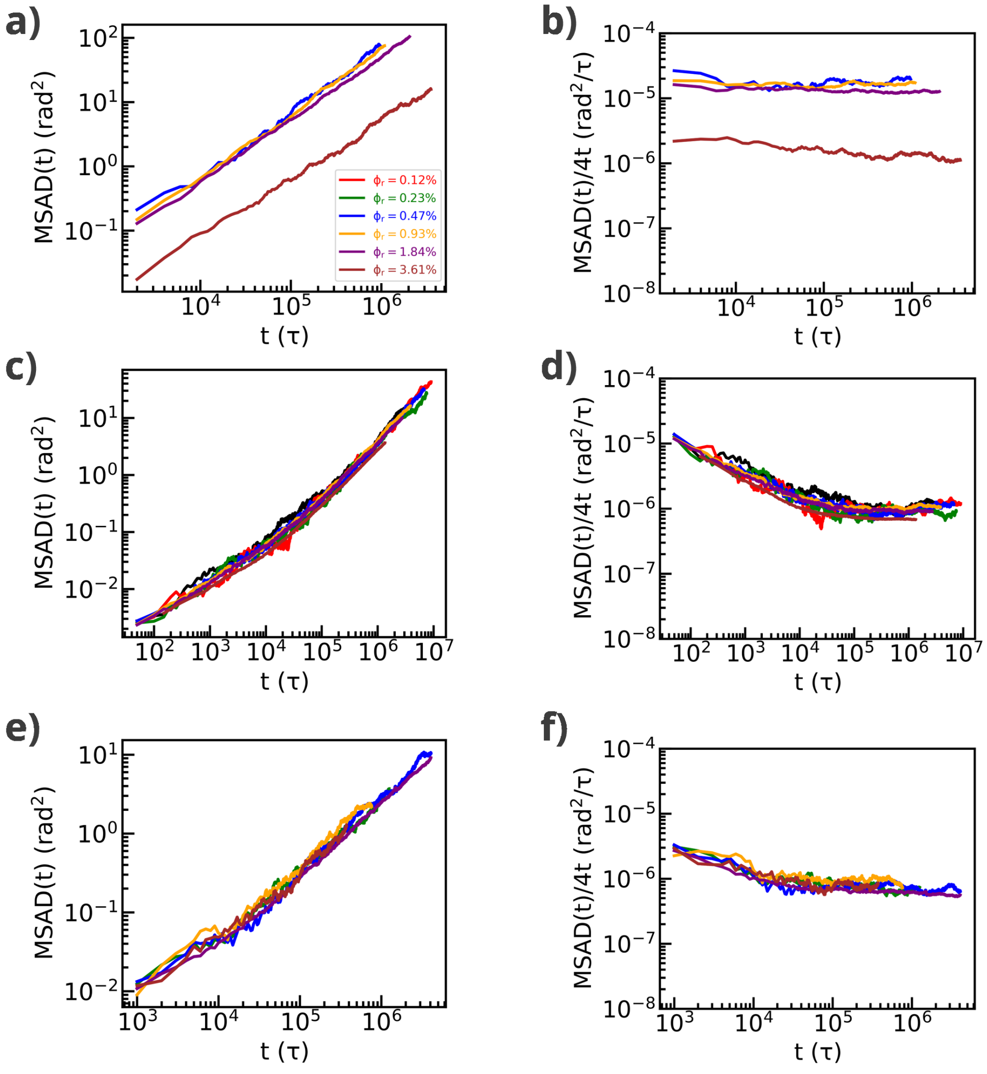

Figure S4: Mean squared angular displacements $MSAD(t)$ and $MSAD(t)/4t$ as a function of nanorod volume fraction ϕ_r for nanorods of length $l = 32\sigma$ in polymer matrices of matrix chain length (a,b) $N = 5$, (c,d) $N = 100$, and (e,f) $N = 400$.

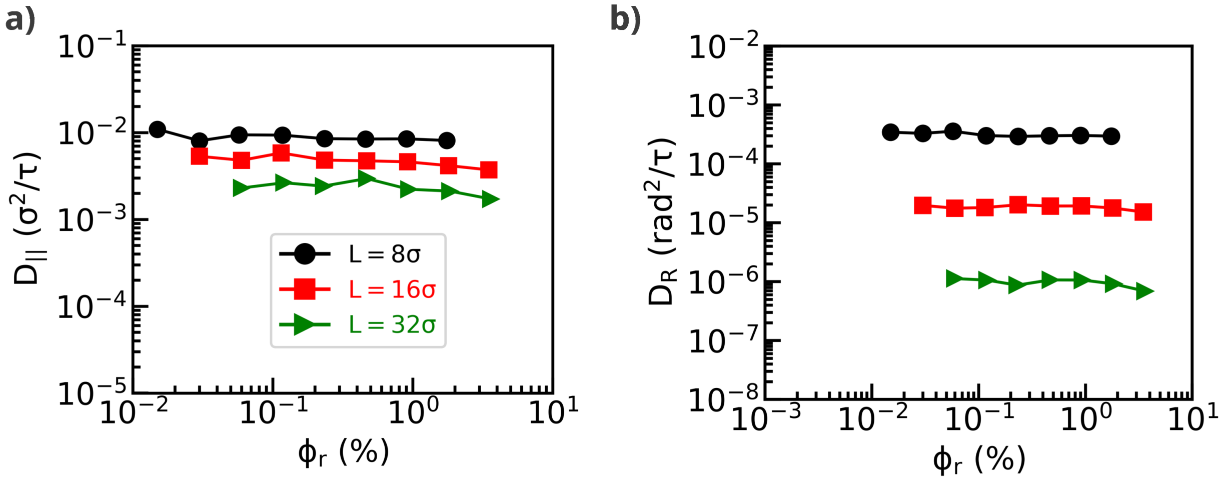

Figure S5: Nanorod diffusion coefficients in polymer matrices of matrix chain length $N = 100$ for nanorods of lengths $l = 8, 16, 32\sigma$ as a function of nanorod volume fraction ϕ_r. Data is shown for (a) Parallel nanorod diffusion coefficients $D_{||}$ and (b) Rotational nanorod diffusion coefficients D_R.
